# Vascular Ensheathment Reflects Characteristic Migratory Behavior of Paragangliomas

**DOI:** 10.1210/jcemcr/luae064

**Published:** 2024-04-15

**Authors:** Leor Needleman, Floyd Christopher Holsinger, Justin P Annes

**Affiliations:** Department of Medicine, Division of Endocrinology, Stanford University, Stanford, CA 94305, USA; Division of Head and Neck Surgery, Department of Otolaryngology, Stanford University, Stanford, CA 94305, USA; Department of Medicine, Division of Endocrinology, Stanford University, Stanford, CA 94305, USA

**Keywords:** paraganglioma, glomus jugulare, DOTATATE

## Image Legend

**Figure 1.**
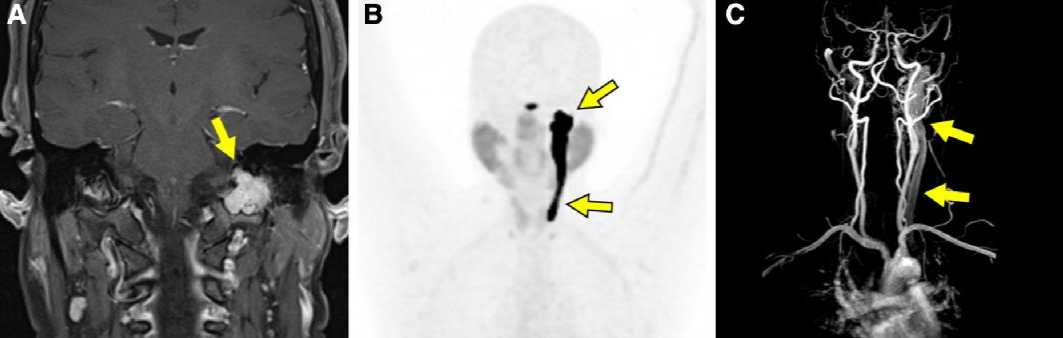
Magnetic resonance imaging, ^64^Cu-DOTATATE positron emission tomography and magnetic resonance angiography of the skull base and neck.

A 43-year-old female patient presented with recurrent left ear infections and left-sided pulsatile tinnitus. Magnetic resonance imaging (MRI) revealed a left jugular fossa mass eroding the skull base consistent with a glomus jugulare (GJ) tumor (Fig. 1A, arrow). Plasma catecholamine metabolites were normal and genetic testing for hereditary paraganglioma was negative. Functional imaging (^64^Cu-DOTATATE positron emission tomography) to assess the extent of primary disease and evaluate for metastasis showed marked uptake in the left jugular foramen with tumor extension along the internal jugular vein (JV) to the level of the thyroid (Fig. 1B, arrows). The caudal tumor extension was not apparent on the initial skull-base MRI. Magnetic resonance angiography showed splaying of the left internal and external carotid arteries and circumferential involvement of the JV (Fig. 1C, arrows). Head/neck paragangliomas can grow within the JV adventitia. In the Fisch classification system of temporal bone paragangliomas, class C tumors spread inferiorly along the JV and are subclassified according to the extent of carotid artery involvement (1). In a surgical series of 16 paragangliomas with JV invasion, GJ tumors were most frequently represented (69%) (2). Functional imaging may be superior to anatomic imaging for visualizing the migratory behavior of paragangliomas along vasculature.

## Data Availability

The data underlying this article are available in the article.

## Abbreviations

GJ: glomus jugulare
JV: jugular vein
MRI: magnetic resonance imaging

## References

[1] Fisch U, Mattox D. Microsurgery of the Skull Base. Thieme; 1988.

[2] Orru’ E, Gursoy M, Gailloud P, et al Jugular vein invasion rate in surgically operated paragangliomas: a multimodality retrospective study. Clin Imaging. 2014;38(6):815‐820. 24908369 10.1016/j.clinimag.2014.04.013

